# Complete Mitochondrial Genome Sequence of Bighorn Sheep

**DOI:** 10.1128/genomeA.00464-18

**Published:** 2018-06-07

**Authors:** Kimberly M. Davenport, Mingrui Duan, Samuel S. Hunter, Daniel D. New, Matthew W. Fagnan, Margaret A. Highland, Brenda M. Murdoch

**Affiliations:** a Department of Animal and Veterinary Science, University of Idaho, Moscow, Idaho, USA; b Institute for Bioinformatics and Evolutionary Studies (IBEST), University of Idaho, Moscow, Idaho, USA; c Animal Disease Research Unit, Agricultural Research Services, USDA, Pullman, Washington, USA

## Abstract

We report here the complete mitochondrial genome sequence of a Rocky Mountain bighorn sheep (Ovis canadensis) in the United States. The circular genome has a size of 16,466 bp and contains 13 protein-coding genes, 22 tRNA genes, and 2 rRNA genes.

## GENOME ANNOUNCEMENT

The bighorn sheep (Ovis canadensis) is an important ecological model for studying natural selection and evolution in western North America (1–4). The population of bighorn sheep drastically declined in the early 20th century due to habitat loss, disease, and overharvest, coinciding with European settlement, but it has substantially rebounded because of conservation efforts and management strategies (5, 6). However, this population decrease led to a bottleneck effect and reduced genetic diversity (4). Investigating genetic diversity and effective population sizes in bighorn sheep will aid in the continued management and conservation of this species (4).

Mitochondrial genetic sequences from many species have been used for population genetics analyses and discerning phylogeny (7–9). Numerous mitochondrial genomes are available for different breeds of domestic sheep (Ovis aries); however, only one has been released for bighorn sheep from Canada (10). Here, we report a complete mitochondrial genome of the Rocky Mountain bighorn sheep from an 8-month-old male in the United States. The animal was raised in a small cohort in captivity at Washington State University in Pullman, WA, under the guidelines of the Institutional Animal Care and Use Committee and Association for Assessment and Accreditation of Laboratory Animal Care.

Mitochondrial DNA (mtDNA) was extracted from the liver with an mtDNA isolation kit (Abcam, Cambridge, MA). Nextera shotgun libraries were produced and sequenced using a v3 600-cycle kit and an Illumina MiSeq instrument by the IBEST Genomics Resources Core at the University of Idaho. Adapter sequences were trimmed, low-quality ends were removed, and pair-end reads were overlapped by HTStream (https://github.com/ibest/HTStream). Cleaned data were assembled by the ARC software package v1.1.4-beta (https://github.com/ibest/ARC) using the Ovis aries isolate GP092 mitochondrial genome (GenBank accession number KF302455) as a seed reference to initialize the iterative assemblies. The assembly resulted in one circular contig, as confirmed by dot plot. The ends were overlapped and joined, and the resulting sequence was linearized such that the orientation started with tRNA-Phe to match other sheep mitochondrial genomes. The complete genome is 16,466 bp, with a GC content of 38.9%. The structural and functional annotation was performed with the mitochondrial genome annotation (MITOS) Web server (11). Annotations of genes were checked using homology searches on GenBank and further improved by manual curation in Geneious v9.1.8 (http://www.geneious.com) (12). The bighorn sheep mitochondrial genome is predicted to have 22 tRNA genes, 2 rRNA genes (12S and 16S), and 13 respiratory genes common to most animal mtDNA (*ATP6*, *ATP8*, *CYTB*, *COX1*, *COX2*, *COX3*, *ND1*, *ND2*, *ND3*, *ND4*, *ND4L*, *ND5*, and *ND6*). Alignment with other sheep mitochondrial genome sequences showed 99.6% identity with that of bighorn sheep and 96% with sequences of domestic sheep, which is 3 million years divergent (2). This suggests that the mtDNA sequence we obtained is consistent with phylogenetic relationships for the studied populations of *Ovis* species. This complete mitochondrial genome provides an additional resource for phylogeographic and population genetic investigations in bighorn sheep, which contributes to future studies on sheep evolution and conservation efforts.

### Accession number(s).

This mtDNA genome sequence has been deposited in GenBank under the accession number MH094035.
